# Exploring lifestyle and dietary pattern shifts among Saudi adults during COVID-19 pandemic: insights from a cross-sectional examination

**DOI:** 10.3389/fnut.2024.1489160

**Published:** 2025-01-06

**Authors:** Najlaa M. Al-Mana, Tahani A. Zareef, Fatmah A. Albathi, Hala A. Awney, Farah Baeshen, Renad Abdullah

**Affiliations:** ^1^College of Applied Medical Sciences, King Saud bin Abdulaziz University for Health Sciences, Jeddah, Saudi Arabia; ^2^Department of Public Health, College of Health Sciences, Saudi Electronic University, Jeddah, Saudi Arabia; ^3^Department of Environmental Studies, Institute of Graduate Studies and Research, Alexandria University, Alexandria, Egypt

**Keywords:** COVID-19, pandemic, lockdown, lifestyle, dietary pattern, Saudi population

## Abstract

**Background:**

Since the emergence of COVID-19 and the subsequent imposition of lockdown and movement restrictions, the world has witnessed fundamental lifestyle changes including alterations in dietary patterns and food consumption habits. Here, we investigated how the COVID-19 lockdown impacted dietary patterns and eating behaviors in the Saudi population.

**Methodology:**

This cross-sectional study enrolled 427 participants aged 18 years or more, with 258 of them completing the survey. The survey included questions about demographic and dietary patterns during the COVID-19 lockdown. Data were collected and dietary behaviors before and during the lockdown in Jeddah, Saudi Arabia, were analyzed.

**Results:**

The number of participants who considered lunch as their primary meal significantly decreased (*p* < 0.001) during the COVID-19 lockdown (74%), compared to before it (86%). By contrast, the number of participants who considered dinner as their primary meal remained almost unchanged (*p* = 0.079) during (79.1%) and before (84.1%) the lockdown. However, snack consumption significantly increased (*p* < 0.001) while fast-food consumption significantly decreased (*p* < 0.01) during the lockdown period. Our results also revealed a significant increase (*p* < 0.01) in water and coffee intake during the lockdown, with a significant rise in dessert consumption (*p* < 0.01).

**Conclusion:**

Our results demonstrate that the COVID-19 lockdown caused a marked shift in dietary patterns and eating behaviors among the Saudi population. Notable changes were observed in overall food preferences after the lockdowns were imposed, with reduced consumption of fast foods and increased fluid intake.

## 1 Introduction

In the last quarter of 2019, a virus with unknown aetiology was identified and initially named 2019 novel coronavirus (2019-nCoV virus), later renamed as SARS-CoV-2 (1–3). In January 2020, the World Health Organization (WHO) called the viral infection coronavirus disease 2019 (COVID-19) and announced a public health crisis (4, 5). In March 2020, COVID-19 was declared a pandemic by the WHO and a disease of global concern (6). Millions of people were affected across more than 100 countries, with an estimated incidence of 10% and numerous deaths (7–9).

The COVID-19 pandemic not only presented itself as a health crisis but also restricted free movement and social interaction, leading to social and lifestyle-related challenges (10, 11). Various aspects of daily life, including dietary patterns and eating behaviors, were profoundly changed (12–15). Restrictions such as social distancing, stay-at-home orders, and work-from-home arrangements inevitably influenced food consumption and purchasing habits (13, 16).

Previous studies have reported an increase in food consumption during the pandemic, with a large proportion of respondents indicating changes in their dietary behaviors (12). Additionally, the stress and emotional distress experienced by individuals during the pandemic further influenced their eating habits (12, 13, 16, 17). Although balanced and healthy food habits were paramount to combat the disease, a shift toward unhealthy food patterns and habits was observed, including the consumption of comfort food with high calories and unrestricted eating between major meals (18–21).

The lockdown measures implemented in Saudi Arabia, including restricted movement and limited access to grocery stores, contributed to these dietary and behavioral modifications (13, 16). Moreover, changes in sleep routines and a lack of physical activity also affected food consumption and dietary patterns (12, 22). Research has revealed that a substantial number of people gained weight during the lockdown in Saudi Arabia because of stress, reduced sleep, and decreased physical activity (16, 22). Furthermore, a study reported an increased consumption of snacks, sweets, fruits, and vegetables during the lockdown period (22).

The impact of the COVID-19 pandemic on Saudi university students’ social and educational aspects has been well-documented; however, the economic and behavioral aspects, including changes in dietary patterns and eating habits, remain understudied (23). Thus, further research is needed to understand the multifaceted effects of the pandemic on the Saudi population, particularly concerning dietary habits, food security, and the complex interactions between lifestyle factors, stress, and health outcomes (24). In this study, we examined the effects of the lockdown imposed during the COVID-19 pandemic on the dietary patterns and eating behavior of the population of Jeddah, Saudi Arabia.

## 2 Materials and methods

### 2.1 Study design and participants

This cross-sectional study investigated the impact of the COVID-19 lockdown on dietary patterns and eating behaviors among the Saudi population in Jeddah City. An anonymous online survey was conducted between September and November 2020. The selection criteria were: male or female healthy adults aged 18–70 years, who were in home quarantine with internet access. The exclusion criterion for this study was individuals who worked outside the home during the lockdown period, those with serious health issues or COVID-19 infection, and lactating or pregnant women were excluded. Non-probability convenience sampling was used for data collection due to its cost-effectiveness and easy accessibility during the pandemic quarantine, enabling diverse responses from individuals not typically reached by other sampling methods. A total of 258 healthy adults aged 18–70 were included in the study.

Approval was obtained from the Ethics Research Committee at King Saud bin Abdulaziz University for Health Sciences (H-01-R-005), ensuring adherence to ethical standards. Informed consent was acquired from all respondents before commencing the online survey.

### 2.2 Survey questionnaire

The online survey was designed using Google Forms in Arabic and English. The survey encompassed several sections, starting with demographic information such as age, gender, location, employment status, education level, weight, and height. It also explored dietary and lifestyle patterns, covering aspects such as time spent indoors, weight fluctuations, daily food intake, meal frequency, sedentary behavior, sleep duration, and concerns related to grocery shopping. A thorough review of the relevant literature and consultation with experts in the field were conducted. Furthermore, the questionnaire was tested by a pilot study with volunteers from the target demographic, resulting in refinements and clarifications to the survey items. The survey compared general dietary behaviors before and during the COVID-19 lockdown, including changes in meal numbers, snack types and quantities, and coffee and water consumption, as well as the impact of the lockdown on diet quality and meal timings. To maintain data integrity, measures such as limiting submissions per user, accessing through email only, deletion of duplicated emails, and setting response ranges for accuracy were implemented. The collected data were carefully checked for inconsistencies or errors before inclusion in the final analysis, ensuring their quality and reliability of the obtained data. The current online survey findings were presented in accordance with the Checklist for Reporting Results of Internet E-Surveys (CHERRIES) guidelines (25).

### 2.3 Statistical analysis

Data analysis was conducted using SPSS. A descriptive analysis was performed for all variables, with the quantitative variables being presented as mean ± standard deviation (SD), and the qualitative data reported in terms of frequency and percentage. The Wilcoxon test for categorical variables was used to compare the differences between paired groups. Non-parametric approaches such as the Wilcoxon signed-rank test, the Mann–Whitney *U* test, and the Kruskal–Wallis test were used to examine the association between a continuous variable and a set of categorical variables. The non-parametric approach was selected because of the differences in sample size between different groups and largely non-normally distributed data, per the findings of the Shapiro–Wilk test (all *p*-values <0.05).

## 3 Results

### 3.1 Demographic characteristics

The sample consisted of 258 participants, with 205 females (79.5%) and 53 males (20.5%). The participants had the following demographic and anthropometric characteristics: a mean height of 160.19 ± 10.66 cm, weight of 67.55 ± 23.01 kg, and BMI of 26.54 ± 12 kg/m^2^. Most of them were young adults aged 18–24 years (*N* = 162, 62.8%), followed by those aged 25–34 years (*N* = 32, 12.4%), 35–44 years (*N* = 20, 7.8%), 45–54 years (*N* = 22, 8.5%), 55–59 years (*N* = 9, 3.5%), and 60–66 years (*N* = 2, 0.8%). A substantial number of participants were employed full-time (*N* = 148, 57.4%) and were unmarried (*N* = 182, 70.5%). Almost half of them had obtained a bachelor’s degree (*N* = 125, 48.4%) and lived with their family (96.5%). Table 1 lists the demographic characteristics of the study population.

**Table 1:** Demographic characteristics of the study participants (*n* = 258).

Characteristics	Frequency, *n* (%)	Mean ± standard deviation
Gender		
Female	205 (79.5)	
Male	53 (20.5)	
Age (year)		
18–24 year	162 (62.8)	
25–34 year	32 (12.4)	
35–44 year	20 (7.8)	
45–54 year	22 (8.5)	
55–59 year	9 (3.5)	
60–66 year	11 (4.3)	
Nationality		
Saudi	231 (89.5)	
Non-Saudi	27 (10.5)	
Education level		
Middle school	6 (2.3)	
High school	103 (39.9)	
Diploma	12 (4.7)	
Bachelor’s degree	125 (48.4)	
Master’s degree	8 (3.1)	
PhD degree	4 (1.6)	
Employment		
Student	148 (57.4)	
Employed full-time	35 (13.6)	
Employed part-time	10 (3.9)	
Unemployed	39 (15.1)	
Self-employed	6 (2.3)	
Retired	20 (7.8)	
Marital status		
Single	169 (65.5)	
Married	85 (32.9)	
Separated	1 (0.4)	
Divorced	3 (1.2)	
Height (cm), mean (SD)		160.19 ± 10.7
Weight (kg), mean (SD)		67.55 (23.01)
Body mass index (kg/m^2^), mean (SD)		26.54 ± 12

### 3.2 Dietary patterns

Concerning habits, 46.5% of the participants reported eating more during the night, 19% responded that they ate more during the morning, and 34.5% had no specific time when they ate the most (Table 2). Most (68.2%) participants reported having regular breakfast before the onset of the pandemic and this number changed only slightly during the lockdown period (67.4%). These results were validated by the McNemar test, which indicated no significant differences in breakfast consumption before and during the lockdown (*p* > 0.9). By contrast, a notable decline was observed in the proportion of individuals who identified lunch as their primary meal of the day, with 86% reporting this preference before the lockdown, compared to 74% during the lockdown (*p* < 0.001). In addition, before the COVID-19 lockdown, 79.1% of participants identified dinner as their main meal, and this increased to 84.1% after the lockdown (*p*-value = 0.079). This suggests a trend toward increased dinner consumption, although the result did not reach statistical significance. Collectively, these findings suggest a discernible shift in dietary patterns, characterized by a preference for increased evening consumption relative to midday meals.

**Table 2:** Dietary patterns before and during the COVID-19 lockdown (*n* = 258).

Dietary patterns	Before the COVID-19 lockdown *n* (%)	During the COVID-19 lockdown *n* (%)	*p*-value
Meal’s consumption			
Breakfast	176 (68)	174 (67.4)	0.912
Lunch	222 (86)	191 (74)	**<0.001**
Dinner	204 (79.1)	217 (84.1)	0.079
Consumption of snacks			
Dessert	116 (45)	151 (58)	**<0.001**
French fries	31.4	39.5	**0.011**
Chips	51.6	54.3	0.489
Chocolate	153 (59.3)	156 (60.5)	0.815
Ice cream	94 (36.4)	116 (45)	**0.006**
Nuts	119 (46.1)	134 (51.9)	0.067
Popcorn	92 (35.7)	122 (47.3)	**<0.001**
Other	16 (6.2)	19 (7.4)	0.648
Snacks consumed per day			
0 snacks	35 (14.7)	29 (12.2)	0.095
1 snack	94 (39.5)	47 (19.7)	**<0.001**
2 snacks	70 (29.4)	74 (31.1)	0.745
3 snacks	23 (9.7)	51 (21.4)	**<0.001**
More than 3 snacks	15 (6.3)	37 (15.5)	**<0.001**
Fast-food consumption per week			
Never	20 (8.4)	89 (37.4)	**<0.001**
Once	120 (50.4)	81 (34)	**0.002**
3 times	97 (40.8)	67 (28.2)	**<0.001**
More than 3 times	1 (0.4)	1 (0.4)	0.655

Abbreviations: Frequency (n) and Percentage (%). Values in bold indicate statistical significance at the *p* < 0.05 level.

**Figure 1:**
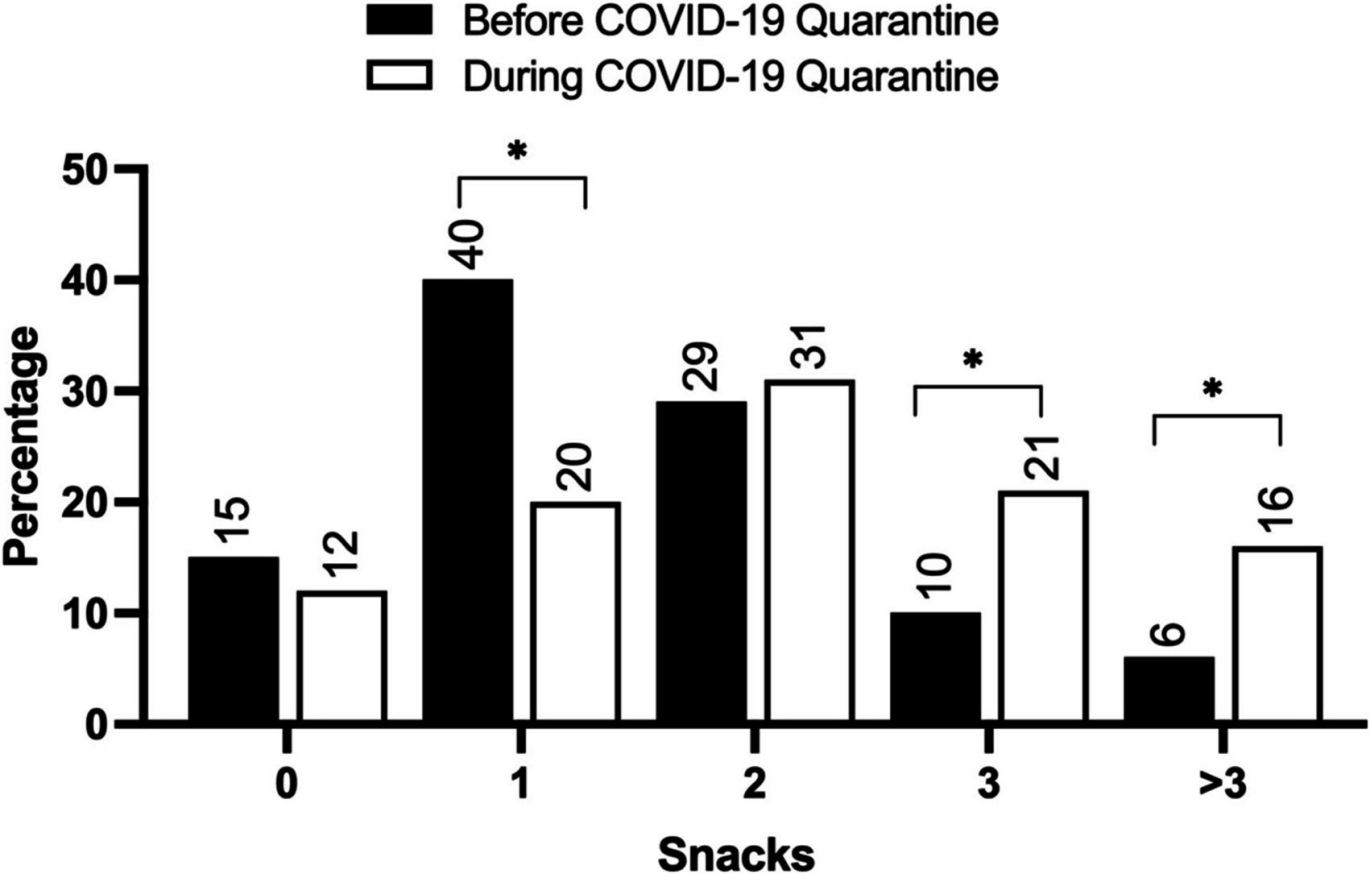
The frequency of snack consumption before and during the COVID-19 lockdown. The values are expressed as the percentage of participants. *N* = 238. ^*^Indicates significance at *p* < 0.05.

We then examined the frequency of snack consumption before and during the COVID-19 lockdown (Figure 1). The results of the Wilcoxon signed-rank test revealed a significant increase in the average number of snacks consumed per day during the lockdown (*M* = 3.03) compared to the pre-lockdown time (*M* = 2.46), with a *Z* statistic of −7.14 and *p* < 0.001. This demonstrates a significant shift toward increased snack consumption during the lockdown period (Figure 1). Furthermore, we observed variations in snack and fast-food consumption. For instance, there was a significant increase in the consumption of desserts during the COVID-19 lockdown (before = 45%, after = 58%, *p* < 0.01), French fries (before = 31.4%, after = 39.5%, *p* = 0.011), ice cream (before = 36.4%, after = 45%, *p* < 0.01), and popcorn (before = 35.7%, after = 47.3%, *p* < 0.01). Additionally, there was a trend toward increased consumption of nuts (before = 46.1%, after = 51.9%, *p* = 0.067), although this did not reach statistical significance. However, the consumption of chocolate, chips, and other types of snacks was not significant and remained consistent before and during the lockdown period (*p* > 0.1).

**Figure 2:**
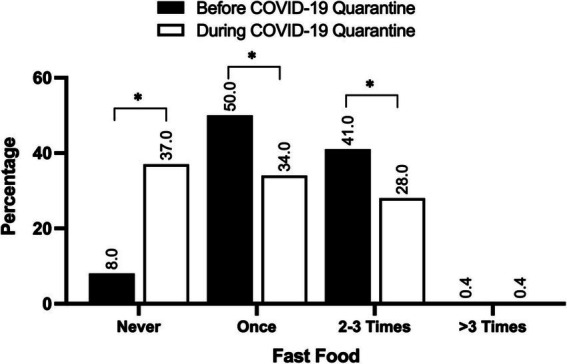
The frequency of fast-food consumption before and during the COVID-19 lockdown. The values are expressed as the percentage of participants. *N* = 238. ^*^Indicates significance at *p* < 0.05.

On the other hand, the Wilcoxon signed-rank test revealed a decrease in fast-food consumption during the lockdown (mean score = 1.92), compared to the pre-lockdown period (mean score = 2.30). This change was significant (*p* < 0.01), demonstrating the robustness of the observed difference. The decrease in fast-food consumption could be attributed to various factors, such as reduced access to fast-food outlets because of the lockdown restrictions, increased awareness of health and nutrition, and changes in household routines and eating habits (Figure 2).

**Figure 3:**
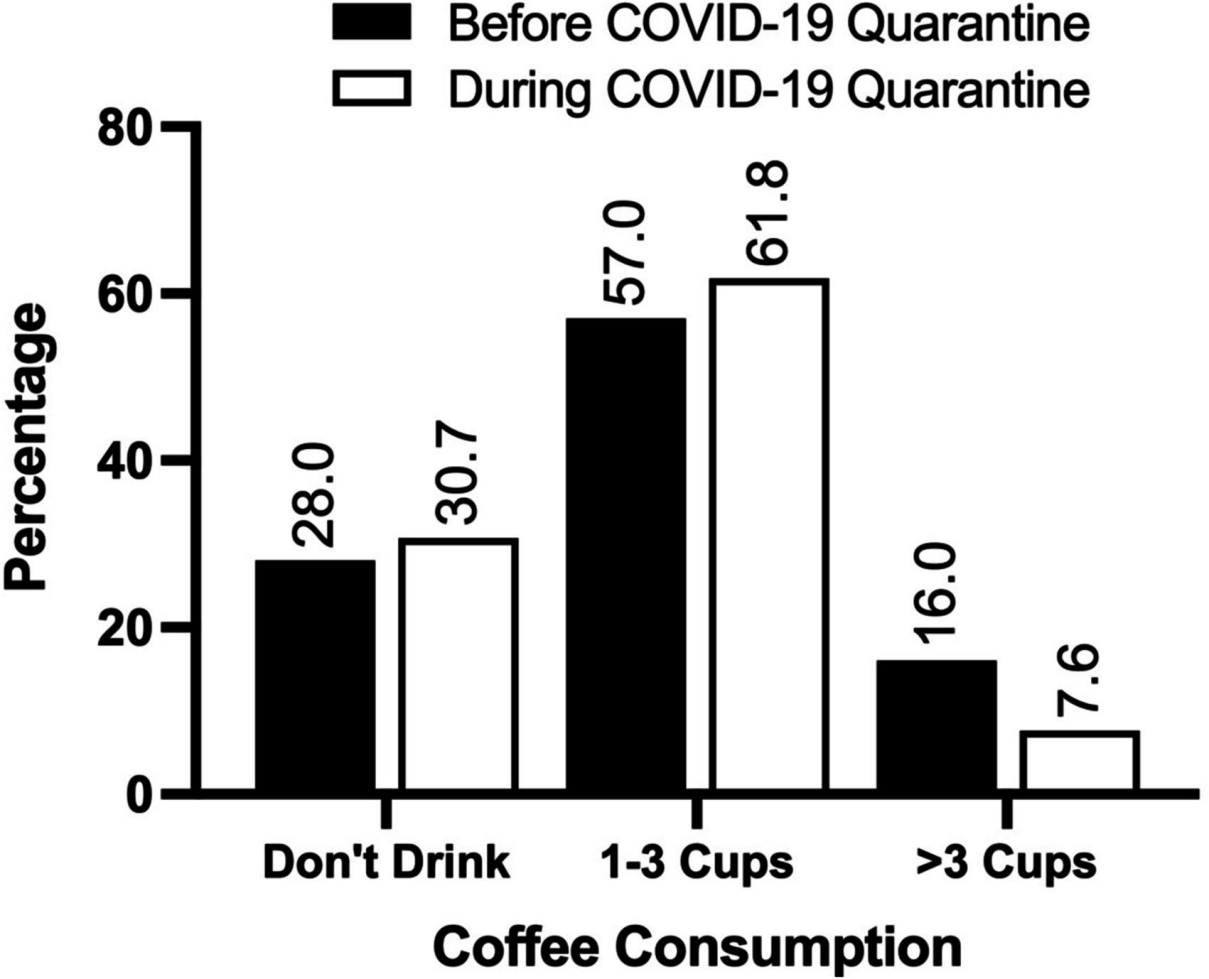
The frequency of coffee consumption before and during the COVID-19 lockdown. The values are expressed as the percentage of participants. *N* = 238.

**Figure 4:**
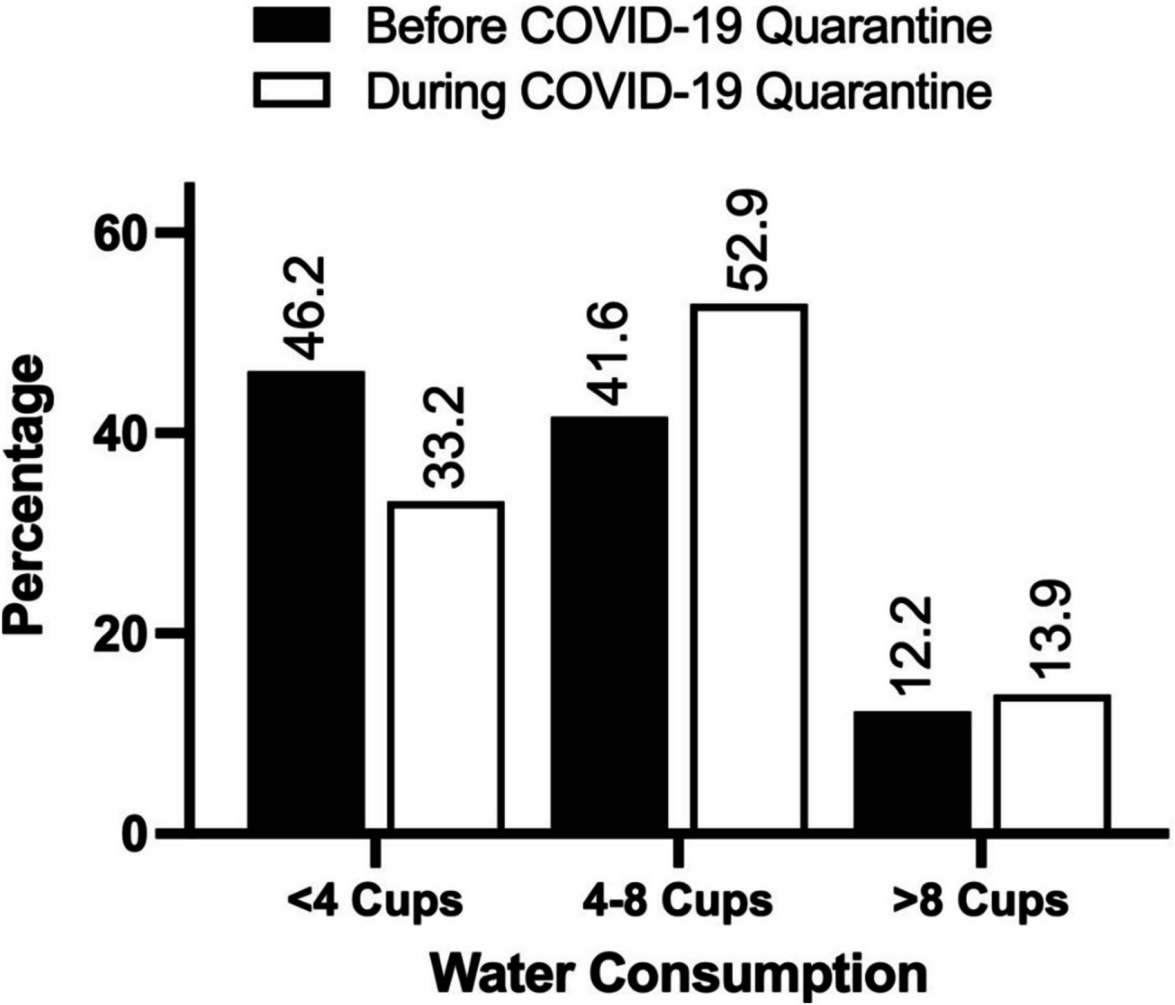
The frequency of water consumption before and during the COVID-19 lockdown. The values are expressed as the percentage of participants. *N* = 238.

Our results showed that the participants’ coffee intake was slightly higher during the lockdown times (*M* = 1.86) compared to before the lockdown (*M* = 1.78), *Z* = −2.64, *p* < 0.01. Similarly, participants reported an increase in water intake during the lockdown (*M* = 1.82) compared to pre-onset intake (*M* = 1.67), *Z* = −3.53, *p* < 0.001 (Figures 3, 4).

### 3.3 Lifestyle patterns of the participants before and after the lockdown

A large proportion of participants reported spending a considerable amount of time sitting, whether for work, leisure, or other reasons, indicating a sedentary lifestyle. A total of 65 participants (25.2%) answered that they spent sitting 4 h or less per day, whereas 105 participants (40.7%) reported sitting for 5–8 h, 57 (22.1%) indicated sitting for 9–11 h, and additional 31 participants reported sitting for more than 11 h per day (12%). This high level of sedentary behavior is concerning given its association with various adverse health outcomes, including increased risk of cardiovascular diseases, obesity, and metabolic syndrome.

Regarding sleep patterns, the study revealed a slight but consistent increase in the number of hours participants slept during the lockdown. The mean value for sleep duration during the lockdown was 2.88 h, compared to 2.59 h before the lockdown. This difference in sleep duration was found to be statistically significant (*Z* = −5.09, *p* < 0.001) based on the Wilcoxon signed-rank test.

### 3.4 Anxiety levels and fear of grocery shopping during the COVID-19 lockdown

The participants exhibited varying levels of anxiety during the lockdown. Approximately 26% of participants reported feeling constantly anxious about the COVID-19 situation, whereas 52.3% reported experiencing anxiety occasionally. On the other hand, 56% of the participants reported never feeling anxious. Among those who felt anxious, always or sometimes, 37.6% attributed their anxiety to their eating habits, while 48% had other reasons. For feeling anxious.

Regarding grocery shopping, a notable percentage of participants reported feeling fearful of grocery shopping; however, most (61.6%) did not experience fear while grocery shopping. Among the participants, 21.3% reported feeling fear and 17.1% struggled to assess whether they experienced fear. This suggests that grocery shopping, which is an essential task during the lockdown evoked fear or uncertainty in a substantial proportion of individuals. Overall, these results highlight the impact of sedentary lifestyles, changes in sleep patterns, and the prevalence of anxiety during the COVID-19 lockdown.

## 4 Discussion

The COVID-19 pandemic has had a profound impact on healthcare systems worldwide, with a cascading effect on other aspects of human life (26–28). The global emergency declaration and the subsequent imposition of lockdowns, travel restrictions, and quarantine wreaked havoc on social, personal, and professional lives throughout the world (29, 30). Major crises such as COVID-19 can disrupt the food system (31) and result in marked changes to individual food preferences and eating behaviors (19). Therefore, this study investigated how the COVID-19 lockdown impacted lifestyle behaviors, especially dietary patterns, in Jeddah, Saudi Arabia.

Our results provide valuable information about lifestyle behaviors, dietary choices, and stress management during the COVID-19 lockdown. The observed shifts in dietary patterns, with a preference for increased evening consumption and snacking, suggest a potential disruption in the participants’ typical eating habits, which could have implications for their overall health and well-being (32, 33). The increase in sedentary behavior and the slight but significant increase in sleep duration may also indicate adaptations to the altered social and environmental conditions imposed by the lockdown. These changes in lifestyle patterns might have contributed to the increased stress and anxiety levels reported by a substantial proportion of the participants.

The changes in dietary preferences and lifestyle behaviors described here are consistent with previous studies. For instance, AlMughamis et al. (34) conducted a study in Kuwait and found a noticeable increase in behaviors such as binge eating episodes, nighttime food consumption, and excessive snacking. A cross-sectional study conducted in the United Arab Emirates reported a notable shift in the dietary patterns of participants experiencing quarantine (35). Other studies also observed unfavorable dietary behaviors during the lockdowns, such as higher caloric intake, more frequent snacking, decreased consumption of fresh fruits and vegetables, and subsequent weight gain (36, 37). Studies conducted by Renzo et al. (38), Ingram et al. (39), Ngoc and Kriengsinyos (40), Mazza et al. (41), Maharat et al. (42), Maharat et al. (43), and Abbas and Kamel (44) also reported important shifts in eating patterns and lifestyle behaviors during the pandemic.

Moreover, our findings are also in agreement with studies that have identified comparable trends to those reported here. For instance, Ammar et al. (18) highlighted increased frequencies of unhealthy food consumption, snacking, and eating in an uncontrolled manner during the pandemic. Similarly, Sidor and Rzymski (45) reported an elevated intake of sweets, snacks, and convenience foods during the pandemic. Additionally, Koçak et al. (46) documented a marked shift in eating habits pre- and post-pandemic, with a decrease in the consumption of fruits, vegetables, legumes, fish, and olive oil, and an increase in the intake of bakery products, sugar or sweeteners, processed meat, and sugar-sweetened beverages.

These collective findings underscore the pervasive impact of the COVID-19 pandemic on dietary preferences and lifestyle choices. The shifts toward nighttime and increased snacking are possible coping mechanisms to address the emotional and psychological challenges induced by the pandemic, including feelings of boredom, isolation, and anxiety. The observed alterations in dietary patterns, such as the increase in snacking and the reduction in fast-food consumption, may represent adaptations by individuals to navigate the stress and uncertainties that resulted from the COVID-19 pandemic and associated lockdowns.

Our results also revealed that coffee and water intake rose during the lockdown. Similar findings about coffee consumption have been reported by other studies which validate the increased coffee consumption in our study (36, 47).

The sedentary lifestyle patterns observed during the COVID-19 lockdown are concerning because prolonged sitting has been associated with various health risks, including cardiovascular disease, obesity, and metabolic disorders. The increased sedentary behavior and sleep patterns observed in this study corroborate previous research. For example, Ingram et al. (39) found a substantial uptick in sedentary behaviors such as watching TV and using the computer during the lockdown period. COVID-19 restrictions hindered physical activity but increased the daily sitting time of the participants (18). Other studies focusing on university students, such as those by Rahman et al. (48), Barkley et al. (49), and Romero-Blanco et al. (50), reported a worsening of sedentary behaviors amidst the lockdown restrictions.

Interestingly, our research also found a slight increase in sleep duration among the participants, which may be linked to reduced commute times and more flexible schedules enabled by remote work and online learning arrangements. This finding is in line with other studies that reported a longer sleep duration during the COVID-19 lockdown. For instance, Cellini et al. (51) reported a slight increase in sleep duration among young adults at the onset of the pandemic. However, while increased sleep duration can be perceived as a positive outcome of the lockdown, prolonged sleep can also be a sign of underlying mental health issues, such as depression, which were exacerbated by the pandemic. Therefore, our data may shed light on this interplay, emphasizing the need to address not just the physical aspects of lifestyle changes but also the mental health implications arising from such shifts.

This study, which examined the psychological impact and lifestyle changes prompted by the COVID-19 lockdown, yielded several notable findings. First, it revealed high anxiety levels among the participants, echoing a growing body of literature that highlights the detrimental mental health repercussions brought about by the pandemic and associated lockdown measures. Witteveen et al. (52), Rajkumar (53), and Son et al. (54) have emphasized the profound negative psychological effects experienced by individuals during this global crisis. In terms of psychological impact, a large portion of our participants reported experiencing anxiety and fear directly associated with the COVID-19 pandemic. This observation aligns with prior research that consistently mentions elevated levels of stress, anxiety, and depression during this challenging period (55).

Moreover, the findings regarding the fear associated with grocery shopping shed light on the broader psychological impact of the pandemic, highlighting the need for targeted interventions to address these concerns and support individuals in maintaining their well-being during challenging times. These findings underscore the importance of developing comprehensive strategies to address the multifaceted challenges posed by the COVID-19 pandemic, encompassing not only physical health but also mental well-being and lifestyle-related behaviors.

Our study has important implications for public health policies and interventions post-COVID-19. The observed changes in lifestyle behaviors, coupled with the heightened anxiety and fear associated with grocery shopping, underscore the multifaceted impact of the COVID-19 pandemic on individuals’ overall well-being. The documented shifts in dietary patterns, physical activity, and sleep habits may have short- and long-term implications for physical and mental health, suggesting the need for comprehensive strategies to support individuals in maintaining a balanced and healthy lifestyle during such challenging times.

## 5 Conclusion

Our results reveal a significant impact of the COVID-19 pandemic and associated lockdowns on individual lifestyles, dietary behaviors, and stress levels. The observed changes, coupled with the prevalence of anxiety and fear, highlight the need for targeted interventions and public health strategies to support individuals in maintaining healthy behaviors during difficult times. Addressing the fears that have influenced purchasing behavior during the pandemic is crucial. This may involve implementing effective strategies to reassure the public and rebuild consumer confidence as we move into the post-pandemic era. Moreover, the shifts in diet habits observed during the crisis raise important questions about their permanence. Understanding whether these changes are temporary or long-lasting is crucial for devising interventions that promote healthier eating habits in the future. Additionally, exploring prevention strategies to avert similar disruptions is imperative. By identifying the underlying factors driving behavioral changes and implementing measures to mitigate their impact, we can better prepare for future crises.

## 6 Limitations and future directions

The present research has several limitations. Recall bias may be present because of the cross-sectional design, limiting the ability to draw causal inferences. Moreover, the reliance on self-reported measures and the convenience approach for collecting data may have led to selection bias. Potential confounding variables such as mental health issues, smoking habits, and physical activity levels should be acknowledged. Exclusion of participants with a history or diagnosis of eating disorders, chronic dieting, or psychological factors, individuals with a history of substance abuse, or those taking medications that could affect cognitive function, potentially impacting eating behaviors and food choices, may considered. By considering these additional confounding factors as exclusion criteria, the study can further ensure the clarity and accuracy of its findings related to the impact of quarantine on dietary patterns and eating behaviors.

Future research should consider controlling for these variables and explore alternative study designs such as longitudinal or experimental studies. Longitudinal designs can reveal the dynamic changes in lifestyle behaviors, dietary patterns, and stress levels throughout the pandemic. Additionally, incorporating objective measures of physical activity, dietary intake, and physiological markers of stress could enable a more nuanced analysis of the impact of lifestyle behaviors on mental well-being during times of crisis.

## Data Availability

The original contributions presented in the study are included in the article/supplementary material, further inquiries can be directed to the corresponding author.
